# Proteolysis Controls Endogenous Substance P Levels

**DOI:** 10.1371/journal.pone.0068638

**Published:** 2013-07-19

**Authors:** Andrew J. Mitchell, Anna Mari Lone, Arthur D. Tinoco, Alan Saghatelian

**Affiliations:** 1 Department of Molecular and Cellular Biology, Harvard University, Cambridge, Massachusetts, United States of America; 2 Department of Chemistry and Chemical Biology, Harvard University, Cambridge, Massachusetts, United States of America; 3 Department of Chemistry, University of Puerto Rico, San Juan, Puerto Rico; Stanford University, United States of America

## Abstract

Substance P (SP) is a prototypical neuropeptide with roles in pain and inflammation. Numerous mechanisms regulate endogenous SP levels, including the differential expression of SP mRNA and the controlled secretion of SP from neurons. Proteolysis has long been suspected to regulate extracellular SP concentrations but data in support of this hypothesis is scarce. Here, we provide evidence that proteolysis controls SP levels in the spinal cord. Using peptidomics to detect and quantify endogenous SP fragments, we identify the primary SP cleavage site as the C-terminal side of the ninth residue of SP. If blocking this pathway increases SP levels, then proteolysis controls SP concentration. We performed a targeted chemical screen using spinal cord lysates as a proxy for the endogenous metabolic environment and identified GM6001 (galardin, ilomastat) as a potent inhibitor of the SP _1–9_-producing activity present in the tissue. Administration of GM6001 to mice results in a greater-than-three-fold increase in the spinal cord levels of SP, which validates the hypothesis that proteolysis controls physiological SP levels.

## Introduction

**Figure 1:**
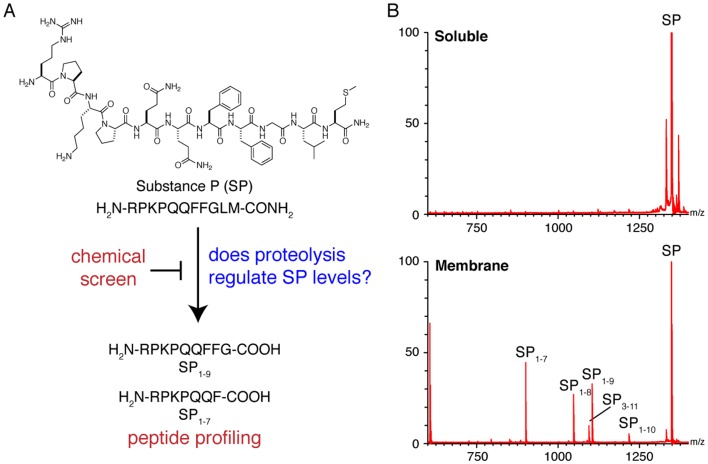
C-terminal processing is the primary mode of SP degradation. A) An integrated approach that combines chemical screening and peptide profiling provides a new strategy to determine whether proteolysis plays a role in the regulation of endogenous SP levels. B) Initial experiments begin in tissue lysates and the data clearly shows that SP is processed by membrane proteases to generate a series of C-terminally truncated fragments, while the soluble proteome has little impact on SP processing.

A member of the tachykinin family of neuropeptides, substance P (SP) is an amidated undecapeptide (Fig. 1) that is widely expressed in the central and peripheral nervous systems [1] of mammals and functions as a neurotransmitter and neuromodulator [2]. It participates in a host of fundamentally and biomedically important physiological processes, including pain transmission [3]–[5], inflammation [6], [7], sleep [8], learning and memory [9], [10], depression and affective mood disorders [11]–[13], opioid dependence [14]–[16] and apoptosis [17], [18]. This broad function profile has driven interest in uncovering the mechanisms that control SP's activity.

Several mechanisms have been definitively shown to regulate SP. These include the differential expression of SP mRNA [19]–[21] and the controlled release of SP from neuron terminals [22]. In view of the well-established role of proteolysis in regulating the activity of certain other bioactive peptides, such as GLP-1 [23] and PHI-27 [24], researchers have postulated that proteolysis of SP in the extracellular space also controls SP levels. A multitude of *in vitro* and pseudo *in vivo* studies indicate SP-degrading activity is abundant in mammalian nervous tissue lend plausibility to this hypothesis [25], [26]. However, one cannot conclude from the mere presence of SP-degrading activity in SP-containing tissues that proteolysis controls SP levels because it is possible that the enzymes responsible for the observed activities do not physically contact endogenous SP in the cell or are otherwise prevented from acting on the peptide (e.g., through protein-protein interactions that are not recapitulated in the test tube). Furthermore, even if one or more of the observed activities acts on SP *in vivo*, it doesn't follow that they control the peptide's levels: the cleavage events may destroy only a small fraction of the total SP present in the extracellular space and therefore not appreciably impact SP concentration. Thus, metabolic studies are not sufficient to establish proteolysis as a mechanism of SP regulation.

One way to definitively determine that proteolysis regulates SP would be to block a proteolytic pathway *in vivo* and show that SP levels change as a result. With this goal, many researchers have sought to identify the enzymes responsible for the SP-degrading activities observed in the aforementioned *in vitro* studies [27]–[33], the idea being that targeted pharmacological or genetic knockdown studies could then be used to probe an SP-degrading pathway. However, to date, no enzyme has been proven to degrade SP *in vivo* and no studies have shown that blocking a proteolytic pathway can modulate SP levels.

Recognizing that enzyme identification approaches are very time consuming given their reliance on extensive biochemical purification and confirmatory studies, we wondered whether there is an easier path to evaluating the hypothesis that proteolysis regulates SP. To this end, we devised a strategy that couples *in vivo* peptidomics with *in vitro* chemical screens to rapidly discover physiologically relevant proteolytic pathways and identify probes that can be used to block them. Focusing our efforts on the spinal cord, where SP executes its most widely studied function of transmitting pain signals from the periphery into the CNS [34], we used this method to determine that a major endogenous SP-degrading pathway cleaves SP at the C-terminal side of residue nine and identify a peptidase inhibitor (GM6001) capable of blocking this pathway. When we injected mice with this compound we observed a greater-than-three-fold increase in endogenous SP levels, thus proving that SP levels are controlled by proteolysis.

## Materials and Methods

### Compounds

Mouse SP was purchased from Anaspec, Inc. A protease inhibitor panel was obtained from Sigma Aldrich Inc.

### Peptide synthesis

Heavy-labeled SP_1–7_ (Pro containing five ^13^C and one ^15^N), SP_1–9_ (Phe containing eight ^2^H), and SP (Leu containing ten ^2^H) were synthesized manually using FMOC chemistry for solid-phase peptide synthesis. Crude peptides were purified by RP-HPLC (Shimadzu) using a C18 column (150×20 mm, 10 μm particle size, Higgins Analytical). The HPLC gradient varied depending on the peptide (Mobile Phase A: 99% H_2_O, 1% Acetonitrile, 0.1% TFA; Mobile Phase B: 90% Acetonitrile, 10% H_2_O, 0.07% TFA). HPLC fractions were analyzed for purity by MALDI-TOF (Waters) using α-cyano-4-hydroxycinnamic acid as the matrix. Pure fractions were combined and lyophilized. Concentrations of the purified peptides were determined by UV-vis using the extinction coefficient for phenylalanine.

### Animal studies

Wild type (C57BL/6) mice used in this study were either purchased (Jackson Labs) or taken from a breeding colony. *Nep^–/–^* mice were obtained from Craig Gerard at Children's Hospital (Boston, MA) and were on a C57BL/6 background [35]. Mice in these studies were not littermates from het x het crosses, but were obtained from separate colonies of *Nep^–/–^* and WT mice. All mice used in this study ranged from 3 to 6 months old. Animals were kept on a 12-h light, 12-h dark schedule and fed *ad libitum.* For spinal cord tissue collection, animals were euthanized with CO_2_, their tissue dissected, flash frozen with liquid N_2_, and stored at −80°C. All animal care and use procedures were in strict accordance with the standing committee on the use of animals in research and teaching at Harvard University and the National Institute of Health guidelines for the humane treatment of laboratory animals. The Harvard institutional Animal Care and Use Committee (IACUC) or ethics committee specifically approved this study and the protocol number is 26–06.

### Isolation of physiological peptides from tissue

Tissue peptide isolation and fractionation were previously described [24], [36], [37]. Briefly, frozen spinal cords were placed in 500 µL of water and boiled for 10 minutes to inactivate any residual proteolytic activity prior to tissue homogenization. The aqueous fraction was separated and saved, and the tissue was dounce-homogenized in ice-cold 0.25% aqueous acetic acid. The aqueous fraction and the homogenate were combined and centrifuged at 20,000×g for 20 min at 4°C. The supernatant was then sent through a 10 kDa molecular weight cut-off filter (VWR Modified PES) to enrich the peptide pool and then a C18 Sep Pak cartridge (HLB 1cc; 30 mg, Oasis) to desalt the sample. The peptides were then eluted with 1 mL of 70:30 H_2_O/ACN and concentrated under vacuum using a speed vac prior to fractionation by strong cation exchange (SCX).

SCX was performed using a PolySULFOETHYL A^TM^ column (200×2.1mm, 5 µm, 300 Å; PolyLC INC.) connected to an Agilent Technologies 1200 series LC. All runs were operated at 0.3 mL/min. The SCX buffers (prepared with MS quality water) consisted of: A) 7 mM KH_2_PO_4_, pH 2.6, 25% ACN (vol/vol); B) 40 mM KCl, 7 mM KH_2_PO_4_, pH 2.6, 25% ACN (vol/vol); C) 100 mM KCl, 7 mM KH_2_PO_4_, pH 2.6, 25% ACN (vol/vol); D) 600 mM KCl, 7 mM KH_2_PO_4_, pH 2.6, 25% ACN (vol/vol). Prior to the SCX runs, all samples (N = 4) were dissolved in 900 µL buffer A (1 mL sample loop). A step-gradient was applied that included 60 min with Buffer A, 60 min with Buffer B, 60 min with Buffer C, and 60 min with Buffer D, with 1 min transitions between the different buffer conditions. Fractions were collected separately for each of the different buffer conditions (e.g., a buffer A fraction, a buffer B fraction, and so on). Fraction C was isolated because SP and the primary peptide products cleaved at the C-terminus are expected to be +3 charged at pH 2.6. This fraction was applied to a C18 Sep Pak cartridge, washed with water to desalt the samples, and then eluted with 1 mL of 70:30 H_2_O/ACN and concentrated using a speed vac. It is important to note that the Met on SP becomes nearly 100% oxidized following SCX. The peptide samples were dissolved in 0.1% aqueous formic acid (50 mg tissue/20 µL), normalized according to the original tissue weight, prior to LC-MS analysis.

### LC-MS/MS experiments to detect SP peptide fragments

Fractionated spinal cord samples (N = 4) were analyzed using a nano flow LC (Nano LC-2D, Eksigent Technologies) system coupled to a linear ion trap mass spectrometer (LTQ, ThermoFinnigan) following a 10 uL injection. The analytical column (Self-pack picofrit column, 75 μm ID, New Objective) was packed 15 cm with 3 μm C18 (Magic C18 AQ 200A 3U, Michrom Bioresources Inc). The trap column was obtained pre-packed from New Objective Inc. (Integrafrit sample trap, C18 5 μm, 100 μm column ID). The samples were trapped at an isocratic flow rate of 2 μl/min for 10 minutes and eluted at a flow rate of 300 nl/min via a mobile phase gradient of 2–50% B in 180 min (Mobile Phase A: 0.1% formic acid in water, Mobile Phase B: 0.1% formic acid in acetonitrile). The peptides were detected in the positive mode and the mass range for data acquisition was set from m/z 400–1600. The data were collected in Top 6 MS2 mode (N = 4) with the dynamic exclusion set for 30s, the exclusion size list set to 200, and the normalized collision energy for CID set to 35%. The capillary spray voltage was set to 2.5 kV. All experiments were repeated multiple times to ensure reproducibility.

Peptide identification was performed via two methods using the SEQUEST algorithm. The first method applied a differential modification of methionine to its sulfoxide. The uniprotmus_frc.fasta mouse database, concatenated to a reversed decoy database, served to estimate a false discovery rate (FDR). Peptides were accepted within 1 Da of the expected mass, meeting a series of custom filters on ScoreFinal (S_f_), −10 Log P, and charge state that attained an average peptide FDR of <2% across data sets. Manual inspection of spectra, FDR calculation, and protein inference were performed in Proteomics Browser Suite 2.23 (ThermoFisher Scientific).

In the second method that has been previously described [24], [37], we used an algorithm written in-house that reveals related MSMS spectra (MuQuest; Harvard Proteomics Browser Suite). To analyze the spinal cord proteome to search for SP fragments, we used the MSMS data from *in vitro* membrane lysate experiments. MuQuest is then applied to compare the *in vitro* MSMS spectra with those of the *in vivo* data set to determine which *in vitro* MSMS spectra (which peptides) are present in the *in vivo* samples. The output files are filtered based on charge state, mass to charge values, and statistical scores. All SP related peptides that were identified in these data were confirmed by analysis of the extracted ion chromatograms (EICs) for these hits.

### Isotope dilution MS (IDMS) to determine endogenous levels of SP and its N-terminal peptide fragments

The heavy-label versions of SP_1–7_, SP_1–9_, and SP were spiked into spinal cord samples (N = 4) at the beginning of the peptide isolation process. After fine tuning the amount of spiked peptides, it was determined that a final concentration of 100 fmol/μL of SP_1–7_ and SP and 25 fmol/μL of SP_1–9_ would lead to adequate measurements of the peptides using Top 6 MS/MS. A comparison was made of the integrated area for specific, corresponding fragments of the +2 charge state of the endogenous and heavy labeled peptides detected in the positive mode. The mass range for data acquisition was set from m/z 400–700. The peptide levels were measured in pmol peptide/g of tissue. All experiments were repeated multiple times to ensure reproducibility.

### Monitoring N-terminal SP fragment formation in spinal cord lysates

Three mouse spinal cords were dounce homogenized in 1.1 mL phosphate buffered saline (PBS) on ice and then sonicated for 15 s at 4°C. Tissue debris was separated by centrifuging the sample at 5,000×g for 20 min at 4°C. The soluble fraction was collected after ultracentrifugation of the sample at 55,000×g for 1 h at 4°C. The membrane pellet that remained was washed 3x with 600 μL PBS. The pellet was then suspended in 100 μL PBS by sonication for 5 s at 4°C. The sample was ultracentrifugation at 55,000×g for 1 h at 4°C and the supernatant was separated as a wash membrane sample. The pellet was suspended in 100 μL of 1 mM sodium deoxycholate (Alfa Aesar) by sonication for 5 s at 4°C then stirred for 30 min at 4°C. Ultracentrifugation of the sample at 55,000×g for 1 h at 4°C separated the supernatant as a 1 mM deoxycholate-solubilized membrane fraction. The remaining pellet was washed 3x with 600 μL 1 mM deoxycholate solution. The pellet was subsequently suspended in 4, 12, and 24 mM deoxycholate following the same cycle. The final pellet was suspended in 100 μL PBS. The protein content in the soluble and membrane lysates was quantified by the Bradford assay. SP (100 μM) was incubated in 1 mg/mL soluble and membrane lysates diluted in 20 mM ammonium bicarbonate, pH 7.34 buffer for 1 h (determined to be the optimal incubation time) at 37°C. The reactions were quenched with neat formic acid and speed vac dried. The samples were dissolved in 0.1% formic acid (aq) and analyzed by MALDI-TOF MS for SP-degrading activity (i.e. formation of SP_1–7_ and SP_1–9_) using the method outlined in “MALDI-TOF MS and LC-MS/MS analysis of *in vitro* peptides” section. All *in vitro* degradation experiments were performed using the same bicarbonate buffer and quenching solutions. Experiments performed with lysates boiled for 5 minutes, served as a negative control.

### Developing a candidate list for the SP degrading enzymes responsible for the formation of SP_1–9_

The MEROPS database was utilized to devise a candidate list for the enzymes that could cleave SP, forming SP_1–9_ in mice [38]. The candidate list was narrowed based on protein abundance using the following databases: www.brain-map.org; https:// www.nextbio.com/b/nextbio.nb.

### Western blotting

Western blotting was used to detect endothelin-converting enzyme 2 (ECE2) (Proteintech Group Inc.; rabbit polyclonal) and pitrilysin (Proteintech Group Inc.; rabbit polyclonal) in the mouse spinal cord membrane lysates prepared by deoxycholate solubilization.

### Protease inhibitor studies using general enzyme class inhibitors

The membrane fraction of spinal cord lysates (1 mg/mL) were pre-incubated at 37°C for 30 minutes separately with each of the following inhibitors (N = 4): 10 μM E-64 (cysteine protease), 1 mM iodoacetamide (cysteine protease), 1 mM o-phenanthroline (metalloprotease), 10 μM pepstatin A (aspartyl protease), 1 mM phenylmethylsulfonyl fluoride (PMSF, serine protease), 1 mM diisopropylfluorophosphate (serine protease), and vehicle (PBS with DMSO for 5% DMSO final concentration in reaction). After the pre-incubation with each inhibitor, SP was added to 100 μM final concentration. The reactions proceeded at 37°C for 1 h. Dried samples were dissolved in 0.1% formic acid (aq) and analyzed by LC-MS/MS for SP-degrading activity (i.e. formation of SP_1–7_ and SP_1–9_) using the method outlined in “MALDI-TOF MS and LC-MS/MS analysis of *in vitro* peptides” section.

### Protease inhibitor studies using metalloprotease specific enzyme class inhibitors

The membrane fraction of spinal cord lysates (1 mg/mL) were pre-incubated at 37°C for 30 minutes separately with each of the following inhibitors (N = 4): SM-19712 (ECE1), phosphoramidon (neprilysin; ECE2), MMP9 inhibitor, TIMP2 (MMP inhibitor), GM6001 (MMP and neprilysin broad inhibitor), chymostatin (pitrilysin), captopril (ACE), enalaprilat (ACE), actinonin (Meprin 1A), and vehicle (PBS with DMSO for 5% DMSO final concentration in reaction). All inhibitors were present at 100 μM except for TIMP2, which was present at 4 μM. After the pre-incubation with each inhibitor, either SP or SP_1–9_ was added to 100 μM final concentration. The reactions proceeded at 37°C for 1 h. Dried samples were dissolved in 0.1% formic acid (aq) and analyzed by LC-MS/MS for SP or SP_1–9_-degrading activity. The same comparative reactions were performed with the deoxycholate-solubilized membrane fractions but only with the GM6001 inhibitor.

### In vivo and in vitro comparative study of SP degradation in WT and *Nep^–/–^* mice spinal cord tissue

The levels of SP in WT and *Nep^–/–^* mice spinal cord tissue (N = 4) were compared by IDMS using Top 6 MS/MS as described previously. The degradation of SP was compared in 1 mg/mL WT and *Nep^–/–^* mice spinal cord membrane lysates using LC-MS/MS analysis following the above method.

### MALDI-TOF MS and LC-MS/MS analysis of *in vitro* peptides

MALDI-TOF MS was performed with α-cyano-4-hydroxycinnamic acid as the matrix using 2 μL of a 50 μM reconstituted degradation reaction solution (based on initial SP quantities). Data were acquired on a Waters MALDI micro MX instrument operated in reflectron positive mode.

For LC-MS analysis, an Agilent 6220 LC-ESI-TOF instrument was used in the positive mode. A Bio-Bond C18 (5 μm, 150×2.1 mm) column was used together with a precolumn (C18, 3.5 μm, 2×20 mm). Following injection of 25 μL of 5 μM solutions, the samples were trapped at an isocratic flow rate of 0.1 ml/min for five minutes and eluted at a flow rate of 0.25 mL/min via a mobile phase gradient of 2–100% B in 40 min (Mobile Phase A: 0.1% formic acid in water, Mobile Phase B: 0.1% formic acid in acetonitrile). MS analysis was performed with an electrospray ionization (ESI) source. The capillary voltage was set at 4.0 kV and the fragmentor voltage to 100 V. The drying gas temperature was 350°C, the drying gas flow rate was 10 L/min, and the nebulizer pressure was 45 psi. Data was collected in the centroid mode using a mass range of 100–500 Da. The peptides were analyzed by mass extraction of the +3 charge state.

### Endogenous GM6001 experiments

For GM6001 injection experiments, 3–4 month old female WT mice (N = 4) were fasted overnight. GM6001 was dissolved at a high concentration in DMSO. Intraperitoneal (i.p.) injections were performed with a 10 μL/g injection of either vehicle (5% DMSO, 95% saline) or 10 mg/mL GM6001 in 5% DMSO, 95% saline for a final dose of 100 mg/kg GM6001. Mice were allowed to return to their cages for three hours and then spinal cords were isolated as described in the ‘Animal studies’ section. IDMS was used to measure differences in the levels of SP in the inhibitor treated and untreated samples.

### Data

All data will be made available upon request.

## Results

### Proteolysis of SP occurs primarily through C-terminal processing

To determine the candidate proteolytic pathway or pathways for SP degradation in the spinal cord, we separated tissue lysates from mouse spinal cords into membrane and soluble fractions, incubated them with full-length SP for varying lengths of time (15, 60, 240 min) and then analyzed the quenched reactions by MALDI mass spectrometry to identify any SP fragments that had been produced. No discernable fragments appeared in the soluble fraction, while ions corresponding to SP_1–10_, SP_1–9_, SP_1–8_, and SP_1–7_ were all produced in the membrane fraction (Fig. 1). These sequence assignments were validated by liquid chromatography-tandem mass spectrometry (LC-MS/MS) [39]. The data indicates that the primary SP-degrading activity in spinal cord resides in the membrane fraction and that proteolysis of SP occurs through C-terminal processing, which is consistent with previous *in vitro* lysate experiments [32], [40], [41].

### SP_1–9_ and SP_1–7_ are endogenous metabolites of SP

Lysate experiments are imperfect because the compartmentalization of a tissue is disrupted, which can lead to the production of SP fragments that are not physiologically relevant. Therefore, we complement these lysate experiments with LC-MS/MS experiments to determine which of these SP fragments, if any, are present in vivo [36]. SP fragment levels are considerably lower than that of full-length SP and therefore we included an offline strong cation exchange (SCX) fraction step prior to the LC-MS/MS to improve our sensitivity [37]. Using this workflow, we are able to detect SP, SP_1–9_ and SP_1–7_ in the spinal cord. We also performed an isotope dilution-mass spectrometry (IDMS) experiment to confirm these results. In these experiments, synthetic stable isotope-labeled versions of SP, SP_1–10_, SP_1–9_, SP_1–8_ and SP_1–7_ are added into the mixture at known concentrations during peptide extraction from spinal cords. These labeled synthetic compounds are easily distinguishable from endogenous SP by mass spectrometry, and the ratio of the stable isotope-labeled peptides to endogenous peptides can be used to calculate the absolute concentration of the SP peptides in the spinal cord. IDMS revealed that the endogenous levels of SP, SP_1–9_ and SP_1–7_ were 105.9±8.5 pmol/g, 2.1±0.5 pmol/g, and 1.6±0.5 pmol/g, respectively (Table 1). We did not detect SP_1–10_ or SP_1–8_ in any of these experiments, suggesting that these peptides are not generated in vivo.

**Table 1:** Absolute quantities of SP and SP fragments in the spinal cord as measured by isotope dilution mass spectrometry (IDMS).

Peptides	SPC (pmol/g)
SP	105.9±8.5
SP_1–10_	n.d.
SP_1–9_	2.1±0.5
SP_1–8_	n.d.
SP_1–7_	1.6±0.5

### The primary SP-degrading enzyme is a metallopeptidase

**Figure 2:**
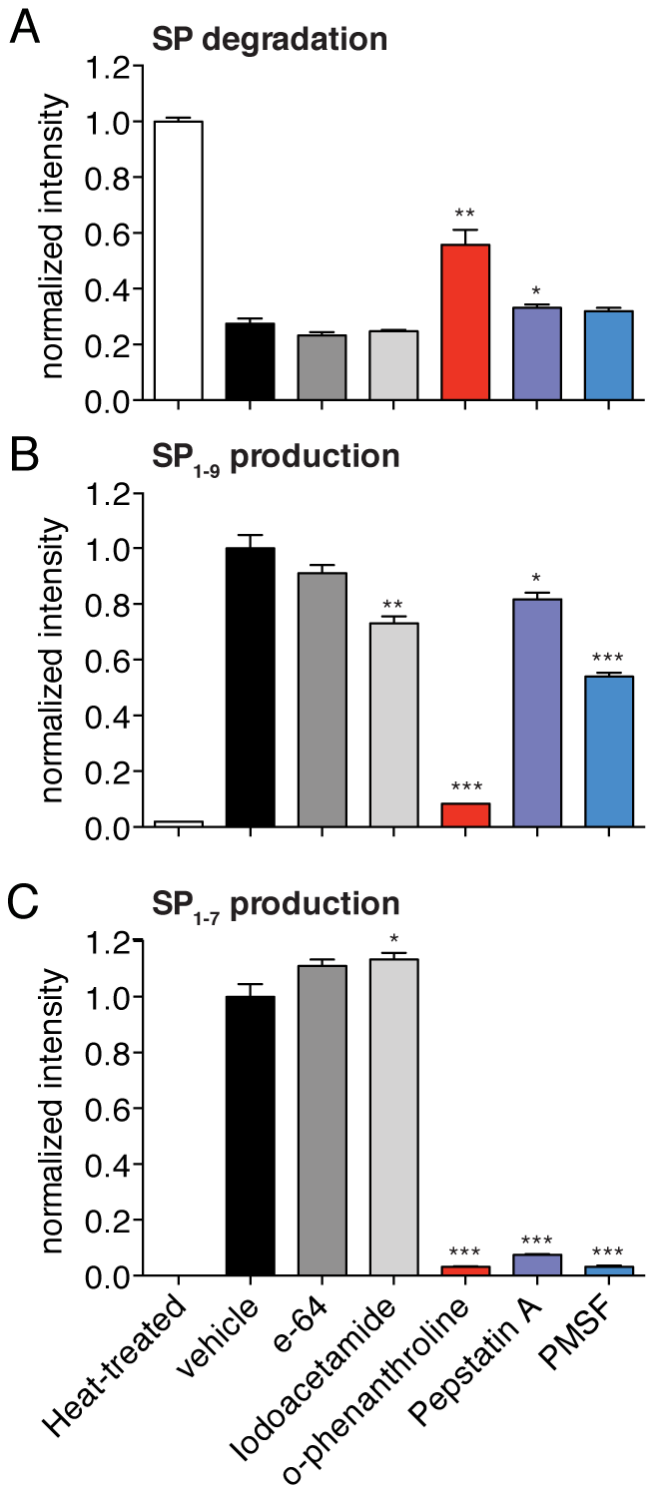
Metallopeptidase inhibitors potently block SP degradation in spinal cord lysates. A) Different class-selective inhibitors were tested for their ability to slow SP degradation in spinal cord membrane lysates. The most effective compound at inhibiting SP degradation in this assay is O-phenanthroline, a metalloprotease inhibitor. B) O-phenanthroline was also the most potent inhibitor of SP_1–9_ production in these experiments. C) Multiple class-selective inhibitors regulate SP1-7 production including O-phenanthroline, pepstatin A and PMSF. (Statistical significance calculated by a Student's t-test; p-value <0.05, *; p-value <0.01, **; p-value <0.001, ***, N = 4).

To identify the enzyme class that mediates SP processing we relied on a screen using class-specific peptidase inhibitors coupled to quantitative MS analysis. The inhibitors used in this screen include compounds such as the metallopeptidase inhibitor O-phenanthroline [42] and PMSF, a serine peptidase inhibitor [43]. We assayed these compounds to test their ability to prevent the SP degradation and inhibit the production of SP_1–9_ and SP_1–7_ in mouse spinal cord lysates (Fig. 2). Lysate samples were heated (i.e. heat-treated) to denature all proteins and this served as a control for complete protease inactivation.

O-phenanthroline inhibited the degradation of SP to the largest extent with SP values twice as high as the vehicle control, indicating that a metallopeptidase contributes the most to SP processing in these tissue lysates. Modest increases in SP levels were also seen for pepstatin and PMSF, but these differences were not statistically significant. O-phenanthroline was also the strongest inhibitor of SP_1–9_ production, and the overall pattern of SP_1–9_ production correlates with the inhibitor specificity for SP degradation. Specifically, O-phenanthroline was the best inhibitor but pepstatin and PMSF had a small effect on SP_1–9_ production. The correlation between SP_1–9_ production and SP degradation indicates that the conversion of SP to SP_1–9_ may be the key step in the conversion of SP in spinal cord lysates. By contrast, the inhibitor sensitivity of SP_1–7_ production is markedly different from that of SP degradation.

### Contribution of reported SP-degrading enzymes to the production of SP_1–9_

Having characterized the conversion of SP to SP_1–9_ as the key step in SP degradation, we turned our attention toward the identification of a chemical inhibitor of this step. SP is often used as a model substrate for peptidases and proteases and therefore there are a number of metallopeptidases reported to cleave SP and produce SP_1–9_ [29], [44]–[48]; however, to our knowledge these enzymes have never been shown to control SP levels in vivo. We reasoned that inhibitors that target these metallopeptidases, even if they are not specific for these proteins, would provide a good chance of finding a compound that could influence SP processing in tissue lysates. The MEROPS database is an authoritative catalogue of all known peptidases and it includes detailed information on peptidase substrate specificity [38].

**Figure 3:**
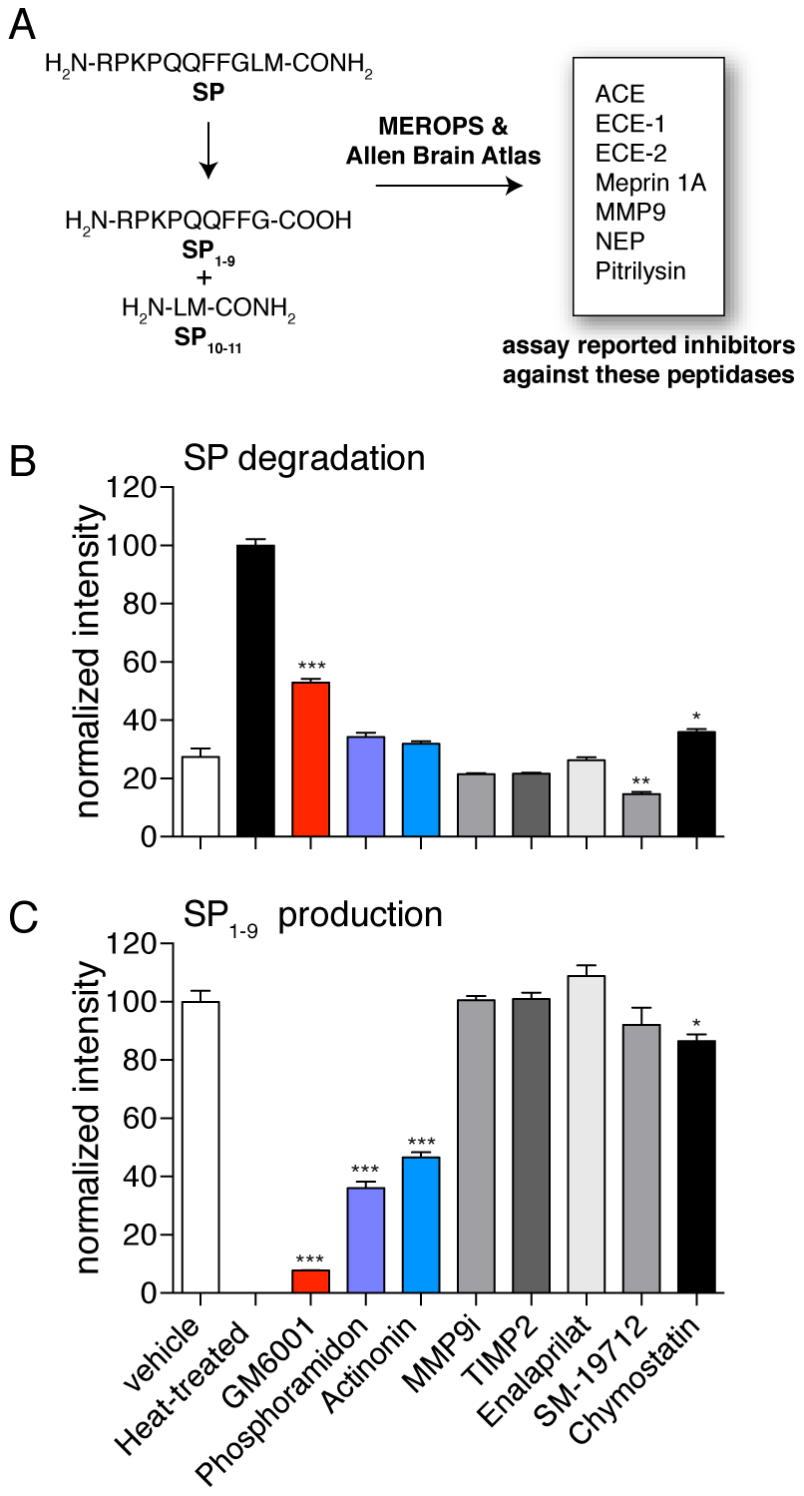
GM6001 is significantly more effective than other metallopeptidase inhibitors at preventing SP degradation and conversion of SP to SP_1–9_. A) Utilizing the MEROPS and Allen Brain Map databases a number of candidate metallopeptidases in the nervous system that are capable of cleaving SP to produce SP_1–9_ are identified. Inhibitors against these peptidases were then used in lysates to evaluate their affect on SP degradation and SP_1–9_ production. B) The matrix metalloprotease (MMP) inhibitor GM6001 was the most effective compound at preventing SP degradation. C) GM6001 is also the best inhibitor of SP_1–9_ production. (Statistical significance calculated by a Student's t-test; p-value <0.05, *; p-value <0.01, **; p-value <0.001, ***, N = 4).

Using the MEROPS database, we identified eight mammalian peptidases reported to cleave SP to produce SP_1–9_ (Fig. 3). These enzymes are all membrane-associated metallopeptidases and are present in the mouse spinal cord [49], [50]. A total of seven metalloprotease inhibitors that target these enzymes were identified with the help of the MEROPS [38] and BRENDA databases [51] (see Materials and Methods). We do not believe any of these inhibitors are selective and therefore we are using these compounds as probes to inhibit the conversion of SP rather than to identify a specific enzyme. For example, the inhibitor actinonin only inhibits meprin 1A [52] but it is also a matrix metalloprotease (MMP) inhibitor, and phosphoramidon inhibits at least two of the candidate enzymes, NEP and ECE-2 (Fig. 3) [53], [54].

Each of these inhibitors was added to spinal cord membrane lysate along with SP. After incubation, the relative levels of SP and SP_1–9_ were assessed in the presence of each inhibitor. This assay revealed three compounds that could inhibit SP degradation and formation of SP_1–9_: phosphoramidon, actinonin and the MMP inhibitor GM6001 [55], [56]. GM6001 led to the least amount of SP degradation and strongly inhibited SP_1–9_ production (Fig. 3). Specifically, addition of GM6001 results in a nearly 2-fold increase in SP levels compared to a vehicle, and a greater than 10-fold decrease in SP_1–9_ production. In aggregate, this data demonstrates that GM6001 inhibits a key step in conversion from SP into SP_1–9_.

**Figure 4:**
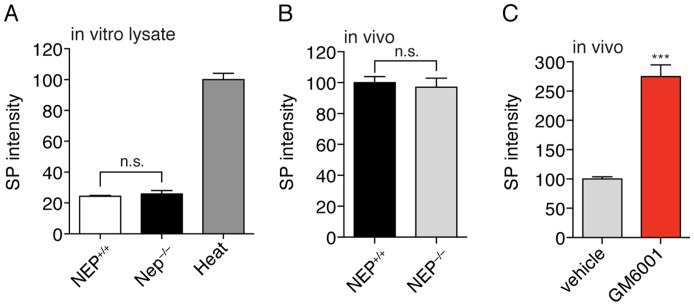
SP is regulated by proteolysis but not by NEP. A) Phosphoramidon slows SP_1–9_ production in tissue lysates, which suggests that NEP might have a role in SP processing. Experiments in NEP^+/+^ and NEP^–/–^ spinal cord lysates reveals no significant difference in SP degradation. B) Likewise, no difference in endogenous SP levels is observed in spinal cords from NEP^+/+^ and NEP^–/–^ mice. C) Acute treatment of mice with GM6001 results in a 3-fold elevation of SP in the spinal cord to reveal a GM6001-sensitive pathway for SP regulation. (Statistical significance calculated by a Student's t-test; p-value <0.001, ***, N = 4).

Based on the inhibitor sensitivities of SP degradation (Fig. 3) NEP and ECE-2 are viable SP-degrading enzyme candidates (Fig. S1). We tested this possibility through several experiments [35]. First, there was no difference in the processing activity between tissue lysates from wild type (NEP^+/+^) and NEP^–/–^ mice [35], indicating that NEP is not the predominant SP-degrading activity in lysates. Furthermore, NEP^–/–^ mice had the same spinal cord concentrations of SP as NEP^+/+^ mice to conclusively demonstrate that NEP does not regulate SP on its own (Fig. 4).

To determine whether ECE-2 is responsible for the SP-degrading activity we detect in tissue lysates, we solubilized the proteome with different concentrations of deoxycholate and then tested these fractions for SP-degrading activity. In addition, we performed activity assays on each of these fractions to ascertain whether ECE-2 levels correlate with SP-degrading activity. These data clearly show that ECE-2 and SP-degrading activity are not connected and this data disfavors any role for ECE-2 in SP-degradation in tissue lysates (Fig. S2). Finally, additional MMP inhibitors (TIMP2 and MMP9i) were not active in protecting SP from degradation either (Fig. 3). These data indicate that none of the likely GM6001 targets is solely responsible for the C-terminal processing and that there may exist a yet unidentified GM6001-sensitive peptidase that regulates SP levels in vivo.

### Small-molecule studies with GM6001 demonstrate that proteolysis regulates SP levels in the spinal cord

Typical approaches for testing enzymes or pathways for a role in peptide regulation *in vivo* require the generation of knockout mice [36]. By using a small-molecule inhibitor approach we circumvent the need for any knockout animals to greatly expedite testing the hypothesis that proteolysis regulates SP levels. Moreover, the use of a small molecule overcomes challenges of compensation by other enzymes that sometimes plague knockout studies [57]. Administration of GM6001 was followed by analysis of SP in the spinal cords after 3 hours. These experiments showed that SP levels were ∼3-fold higher in the mice treated with GM6001 versus the vehicle treated controls (Fig. 4). Thus, we discovered an endogenous GM6001-sensitive proteolytic pathway that regulates SP levels. More importantly, this data confirms the hypothesis that proteolysis controls endogenous SP levels.

## Discussion

The hypothesis that SP is regulated by proteolysis originated with the earliest discoveries of potent SP-degrading activity in nervous tissue, which occurred over three decades ago [27], [58]. Subsequent work identified numerous peptidases [28], [31], [32], [40], [41], [48], [59]–[61] that cleave SP *in vitro*, but none of these enzymes has been shown to regulate SP *in vivo*. Believing that identifying a true SP-regulating enzyme may depend on clearly defining the products of endogenous SP metabolism, many researchers have conducted *in vitro* experiments in which the endogenous metabolic environment of the CNS is simulated [28], [30], [40], [41]. Typically these experiments involve incubating cell lysates with large amounts of synthetic peptide. Others have conducted pseudo-*in vivo* experiments where synthetic peptide is introduced into a living tissue and the excess metabolites are collected and analyzed [62]. In these studies, a multitude of fragments has been detected, among them SP_1–9_, SP_1–8_, SP_1–7_, SP_5–11_, SP_6–11_, SP_7–11_ and SP_8–11_. The data suggests an extremely complicated picture for SP proteolysis and does not present a clear path towards elucidating SP regulation.

Recently, we developed an advanced liquid chromatography-tandem mass spectrometry (LC-MS/MS) peptide profiling strategy to elucidate the proteolysis of bioactive peptides [24], [36]. The power of this approach lies in its ability to peer directly into the chemical milieu in which endogenous metabolism occurs. In applying this approach to SP we circumvent the epistemological worries one may have about the results of *in vitro* and pseudo-*in vivo* studies, which do not perfectly reconstitute endogenous metabolism. We profiled peptides from the mouse spinal cord and detected SP_1–9_ and SP_1–7_, indicating these are endogenous metabolites of SP. We confirmed these assignments and obtained the absolute concentrations of SP, SP_1–9_, and SP_1–7_ in tissues using isotope dilution-mass spectrometry [63]. Our results were consistent with previously reported concentrations of SP in the spinal cord (∼105.9 pmol/g) [64], and demonstrated that SP_1–9_ and SP_1–7_ are approximately 50-fold lower in concentration than SP (Table 1). We also performed *in vitro* degradation experiments and found that enzyme activity capable of producing SP_1–9_ and SP_1–7_ resides in the membrane fraction but not the soluble fraction of spinal cord lysate (Fig. 1). This finding is consistent with the notion that SP processing occurs outside the cell, where the SP is exposed to membrane-bound proteases. The combination of *in vitro* and *in vivo* peptide profiling indicates that SP_1–9_ and SP_1–7_ are the primary endogenous products from processing of SP in the spinal cord.

A series of lysate experiments with class selective inhibitors showed that SP is regulated by a metallopeptidase and that the levels of SP and SP_1–9_ are inversely correlated (Fig. 2). This indicates that SP degradation is coupled to SP_1–9_ production and that the conversion of SP to SP_1–9_ is a major means of SP degradation since the inhibition of this pathway affects SP levels. The results with SP_1–7_ were less clear, as several inhibitors affected SP_1–7_ production and there was no clear correlation between SP_1–7_ production and SP levels. We subjectedSP_1–9_ to degradation in spinal cord lysates and observed that SP_1–7_ was produced (Fig. S3). We reasoned that inhibition of SP_1–9_ production will reduce SP_1–7_ levels and therefore focused on the conversion of SP to SP_1–9_ as the key step in SP catabolism.

At this point, a typical approach would call for the identification of the enzyme responsible for this step followed by perturbation of the protein *in vivo* to verify the identification [36]. However, recognizing that the generation of knockout animals is very slow and often confounded by compensatory effects that mask the function of the enzyme in knockout animals [57] and that knowing the identity of the degrading enzyme is not necessary to evaluate whether SP is regulated by proteolysis, we opted to perform a small screen of metalloprotease inhibitors to identify a compound that could potentially be used to perturb the pathway *in vivo*. In this way we discovered that GM6001 potently blocks the conversion of SP to SP _1–9_in tissue lysates (Fig. 3).

Fortuitously, GM6001 is bioavailable and had previously been shown to permeate the central nervous system (CNS) [65]. This allowed us to test whether GM6001 can influence SP levels in vivo. Administration of GM6001 to mice resulted in a 3-fold increase in SP levels in the spinal cord (p-value <0.001, Fig. 3). While this strategy does not identify any specific enzyme, it quickly reveals whether blocking proteolysis regulates SP levels and proves correct the long-standing hypothesis that SP levels are regulated by proteolysis. Moreover, given this data the enthusiasm for pursuing a SP degrading enzyme is increased and GM6001 can be used as a valuable tool in the discovery of such an enzyme. GM6001 was previously developed into an activity-based probe that targets metalloproteases [66] and this unbiased strategy can be applied to the discovery of a candidate SP-degrading enzyme.

## Conclusions

We expect the strategy described herein to be applicable to all bioactive peptides [67] and we envision the power of the approach increasing with larger chemical libraries and faster high-throughput lysate assays. Thus, this work advances the methodology for the discovery of bioactive peptide regulatory pathways, provides a chemical inhibitor to study SP regulation *in vivo* and conclusively demonstrates that proteolysis plays a major role in the regulation of SP.

## Supporting Information

Figure S1 **Flow chart outlining the strategy used at each step to identify or eliminate a candidate SP-degrading enzyme.** Ultimately this approach identified a GM6001-sensitive pathway in the spinal cord to validate the hypothesis that proteolysis regulates SP levels.
(PNG)

Figure S2 **ECE2 is not responsible for SP-degrading activity in lysates.** Fractionation of spinal cord lysate by successive solvation in the detergent deoxycholate coupled with an LCMS-based assay for SP _1–9_ production shows that ECE2 abundance does not correlate with the activity of interest.
(PNG)

Figure S3 **Existence of a proteolytic activity in spinal cord lysates that converts SP _1–9_ to SP_1–7_. SP_1–7_ is produced when SP _1–9_ is incubated in spinal cord lysate indicating the presence of a SP_1–9_ to SP_1–7_ activity.**
(PNG)
