# Supplementary figures and images for: Proteolysis Controls Endogenous Substance P Levels
Source: PLoS One. 2013 Jul 19;8(7):e68638. doi: 10.1371/journal.pone.0068638 (PMC3716696; doi:10.1371/journal.pone.0068638)

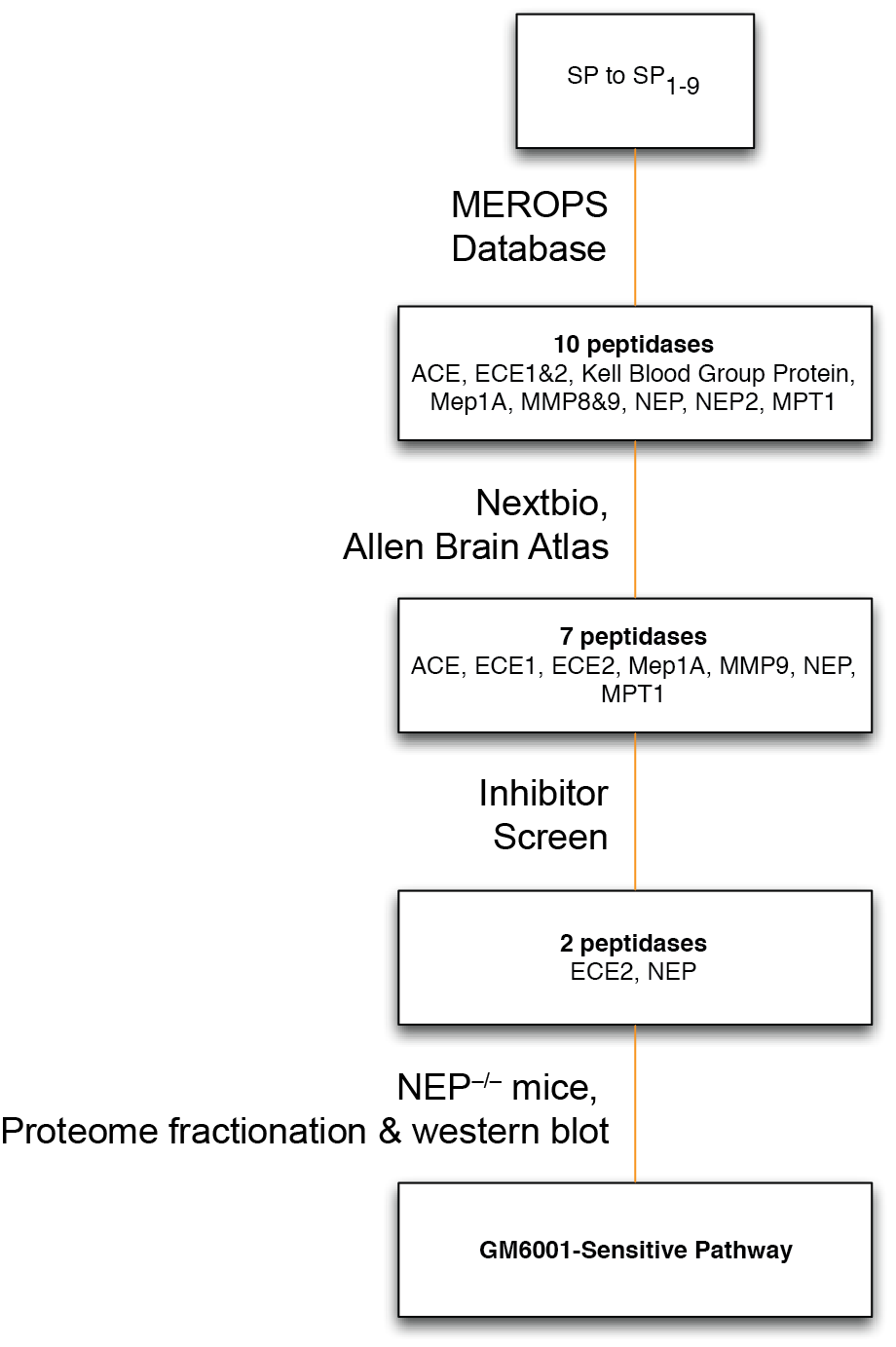

Supplement: Figure S1 — Flow chart outlining the strategy used at each step to identify or eliminate a candidate SP-degrading enzyme. Ultimately this approach identified a GM6001-sensitive pathway in the spinal cord to validate the hypothesis that proteolysis regulates SP levels. (PNG) [file pone.0068638.s001.png]

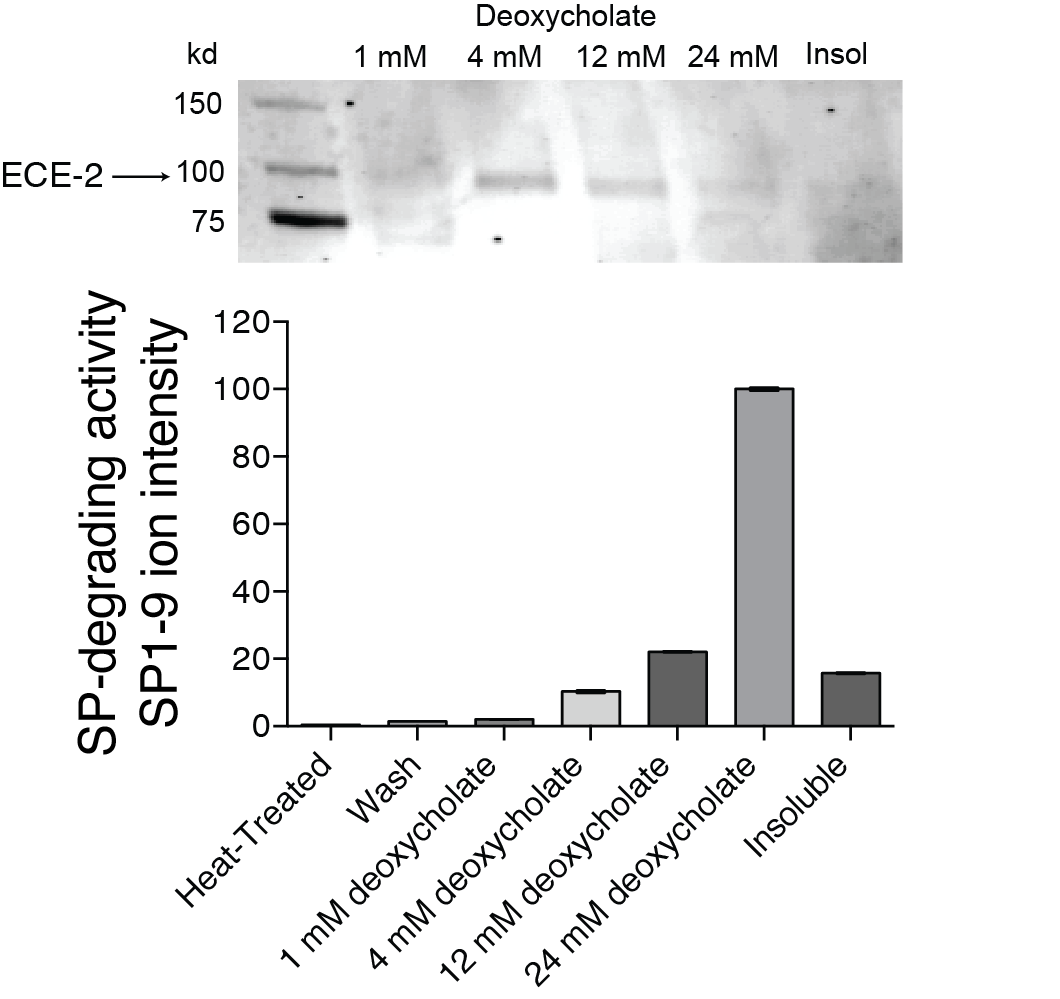

Supplement: Figure S2 — ECE2 is not responsible for SP-degrading activity in lysates. Fractionation of spinal cord lysate by successive solvation in the detergent deoxycholate coupled with an LCMS-based assay for SP 1–9 production shows that ECE2 abundance does not correlate with the activity of interest. (PNG) [file pone.0068638.s002.png]

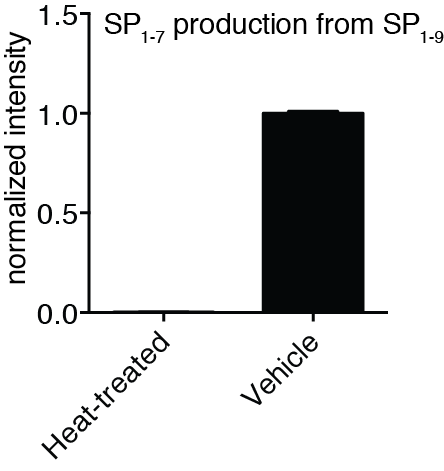

Supplement: Figure S3 — Existence of a proteolytic activity in spinal cord lysates that converts SP 1–9 to SP1–7. SP1–7 is produced when SP 1–9 is incubated in spinal cord lysate indicating the presence of a SP1–9 to SP1–7 activity. (PNG) [file pone.0068638.s003.png]
